# WDR76 regulates 5-fluorouracil sensitivity in colon cancer via HRAS

**DOI:** 10.1007/s12672-023-00656-9

**Published:** 2023-04-20

**Authors:** Yunlong Hu, Xiao Tan, Lin Zhang, Xiang Zhu, Xiangyao Wang

**Affiliations:** 1 Department of Gastroenterology and Hepatology, The First Medical Center of Chinese PLA General Hospital, Beijing, China; 2 Department of Emergency and Intensive Care Unit, The 966th Hospital of Joint Logistic Support Force of PLA, Dandong, China; 3 Center of Medical Security, No. 971th Hospital of Chinese Navy, Qingdao, China; 4 Department of Outpatient Service, No. 986th Hospital Affilliated to Air Force Medical University, Xi’an, China; 5 Army No. 82 Group Military Hospital, Baoding, China

**Keywords:** WDR76, Colon cancer, 5-fluorouracil (5-FU), HRAS

## Abstract

**Background:**

WD repeat domain 76 (WDR76) has been reported in multiple tumors, while without relation to chemotherapy resistance. 5-fluorouracil (5-FU) is widely adopted in treating colon cancer. However, the resistance of WDR76 and 5-FU in colon cancer remains unclear.

**Methods:**

Limma package in R software was employed to analyze the differentially expressed genes. Western blot or quantitative real-time PCR (qRT-PCR) were run to assessed the gene expression. The cytotoxic effect was determined according to cell viability assay, colony formation assay in vitro. Cell apoptosis was assayed using flow cytometry. GSEA analysis was performed to identify pathways related to the target gene. Xenografted mice model was employed to evaluate the tumor growth.

**Results:**

Bioinformatic analysis revealed the higher expression of WDR76 in 5-FU sensitive colon cancer cells compared to resistant colon cancer cells, accompanied by the decreased mRNA expression of WDR76 in 5-FU resistant colon cancer cells. The overexpressed WDR76 resulted in the apoptosis and the downregulated colony numbers in 5-FU resistant colon cancer cells, leading to the elevated sensitivity of 5-FU. Meanwhile, knockdown of WDR76 enhances the resistance of 5-FU in colon cancer both in vitro and vivo, which was reversed by a specific inhibitor of HRAS, Kobe006. An important molecular mechanism of 5-FU resistance lies the degradation of HRAS induced by WDR76.

**Conclusion:**

Our findings demonstrated a role of WDR76 as a promising target for reversing the resistance of colon cancer to 5-FU.

**Supplementary Information:**

The online version contains supplementary material available at 10.1007/s12672-023-00656-9.

## Introduction

Colon cancer is considered a common malignant tumor of digestive tract with high morbidity and mortality, posing a great threat to human health [1–3]. Continuous efforts have been carried out in the diagnosis and treatment of colon cancer. According to the localization, staging of cancer, the clinical condition of patient, pathological classification, metastasis, and clinical stage different types of surgery as well as the different chemotherapy regimens were tailored [4–8]. 5-FU could prevent DNA synthesis of colorectal cancer cells and downregulate the thymidine synthetase activity [9, 10], which has been widely employed to prolong the life time patients with colon cancer [11–13]. However, a long-term use of this drug raises the risk of insensitivity to chemotherapy. The mechanism of 5-FU resistance remains one of the research hotspots in colon cancer.

WDR76 as a member of the WD40 repeats domain superfamily and participates in various biological processes [14], which could be combined with UV damaged DeoxyriboNucleic Acid (DNA), co-expressed with genes related to DNA metabolism, participate in DNA replication stress, and recruited into the coding region to promote transcription [15]. In recent years, it has been confirmed to mediate obesity, liver steatosis and the development of lung adenocarcinoma [16–18]. Moreover, some studies have also proved that WDR76 manipulates the occurrence and progression of hepatocarcinoma by degrading the pan-RAS [19]. However, the mechanism of WDR76 in 5-FU resistance of colon cancer remains unclear.

In the present study, the potential relationship between WDR76 and 5-FU sensitivity of colon cancer was explored and experimentally verified by bioinformatics analysis depending on GEO database, to provide novel biomarkers and/or targets for treatment of colon cancer.

## Materials and methods

### Cell culture

Human derived colon cancer cell HCT116 and COLO320 were all cultured in McCoy’s 5A and DMEM (GIBCO) mixed with 10% fetal bovine serum, 100 IU/mL penicillin and 100 mg/mL streptomycin incubated at 37℃ with 5% CO_2_. Drug resistant cell lines were constructed by adding 5-FU unto HCT116 or COLO320 cells with an increasing concentration until no obvious death was observed.

### Analysis of GEO and TCGA-COAD dataset

GSE157300 and GSE158021 datasets covering gene expression profiles of 6 and 2 DMSO treated samples and 6 5-FU treated samples of HCT116 cells from Gene Expression Omnibus data base (GEO) were employed. RNA-Sequencing data of TCGA-COAD were downloaded from the Cancer Genome Atlas (TCGA) database (https://portal.gdc.cancer.gov/repository). The L imma package in R was employed to analyze the differentially expressed mRNAs (│log2FC│ > 1 and P-value < 0.05). The gene enrichment analysis (GSEA) was performed to analyze the expression of WDR76 and the potential mechanism of tumorigenesis in COAD.

### Plasmid transfection and lentivirus infection

VigoFect or RNA iMAX were employed to transfect plasmid or siRNA into cells, respectively, following the manufacturer's instructions. HCT116 cell line with stably expressed WDR76 shRNA were constructed by infected with the lentiviruses carrying WDR76 shRNA and selected using puromycin. The WDR76 shRNA target sequence referred to 5ʹ-CGCTAAGAAGCCGAAAGATGTCC-3ʹ.

### Quantitative real-time PCR (qRT-PCR)

Trizol was employed to extract the total RNA from colon cancer cells following the manufacturer’s instructions, then transcribed to cDNA using Moloney murine leukemia virus (M-MLV) (Promega). qPCR was run in a reaction mixture for 3 times for each of the primers and cDNA template. Data was normalized according to β-actin, with the result calculated by the comparative Ct method.

### Western blot

RIPA lysis buffer was employed to lyse cells or tumor tissues, which were then separated on SDS-PAGE gels and transferred onto the polyvinylidene fluoride (PVDF) membrane. The primary or secondary antibodies were incubated together with membranes. Antibodies of anti-WDR76 (HPA039804, Sigma-Aldrich; 1:1,000 dilution), anti-β-actin (sc-47778, Santa Cruz Biotechnology; 1:1,000 dilution), anti-Flag (A8592, Sigma-Aldrich; 1:1000 dilution), anti-HRAS (2867S, Cell Signaling Technology; 1:500 dilution), anti-p-ERK (ab229912, ABCAM; 1:500 dilution) and anti-p-MEK (ab96379, ABCAM; 1:500 dilution) were used.

### Colony-formation and apoptosis assays

A total of 3,000 cells were seeded in each 6-well plate and cultured for 14 days for clone formation, then were washed for three times with PBS and fixed with 4% formaldehyde for 20 min, and covered by 0.1% crystal violet solution for 10 min. The colonies in diameter over 1 mm were counted. Cells were dissociated to detect apoptosis rate. Cell apoptosis was measured using Annexin V Apoptosis Detection kit and flow cytometry (BD Biosciences).

### Tumor growth and metastasis analysis in vivo

To evaluate the proliferation of tumor cells in vivo, a total of 1 × 10^7^ HCT116-R cells were inoculated under the skin of left flanks of 6-week female nude mice (n = 6). After 7 days of injection, mice were treated with 5-fluorouracil in every 3 days. Tumor tissues were evaluated at the certain times. All the tumor tissues were conserved in liquid nitrogen after morally killing the mice. All these animal experiments were performed by the Ethics Committees of Chinese PLA General Hospital. The maximal tumor size/burden was not exceeded the permissible tolerance in IACUC protocol (< 2 cm).

### Statistical analysis

All from at least three times independent experiments were expressed as the mean ± SD deviation, which were analyzed by GraphPad 9.0 (CA, USA) and SPSS 25.0 (IL, USA). A two-tailed Student’s test was performed to determine the statistically significance between two groups. ANOVA was employed to determine the statistically significance among three groups or more. A p value of less than 0.05 was considered statistically significant.

## Results

### WDR76 is screened and verified in 5-FU resistant colon cancer

**Fig. 1:**
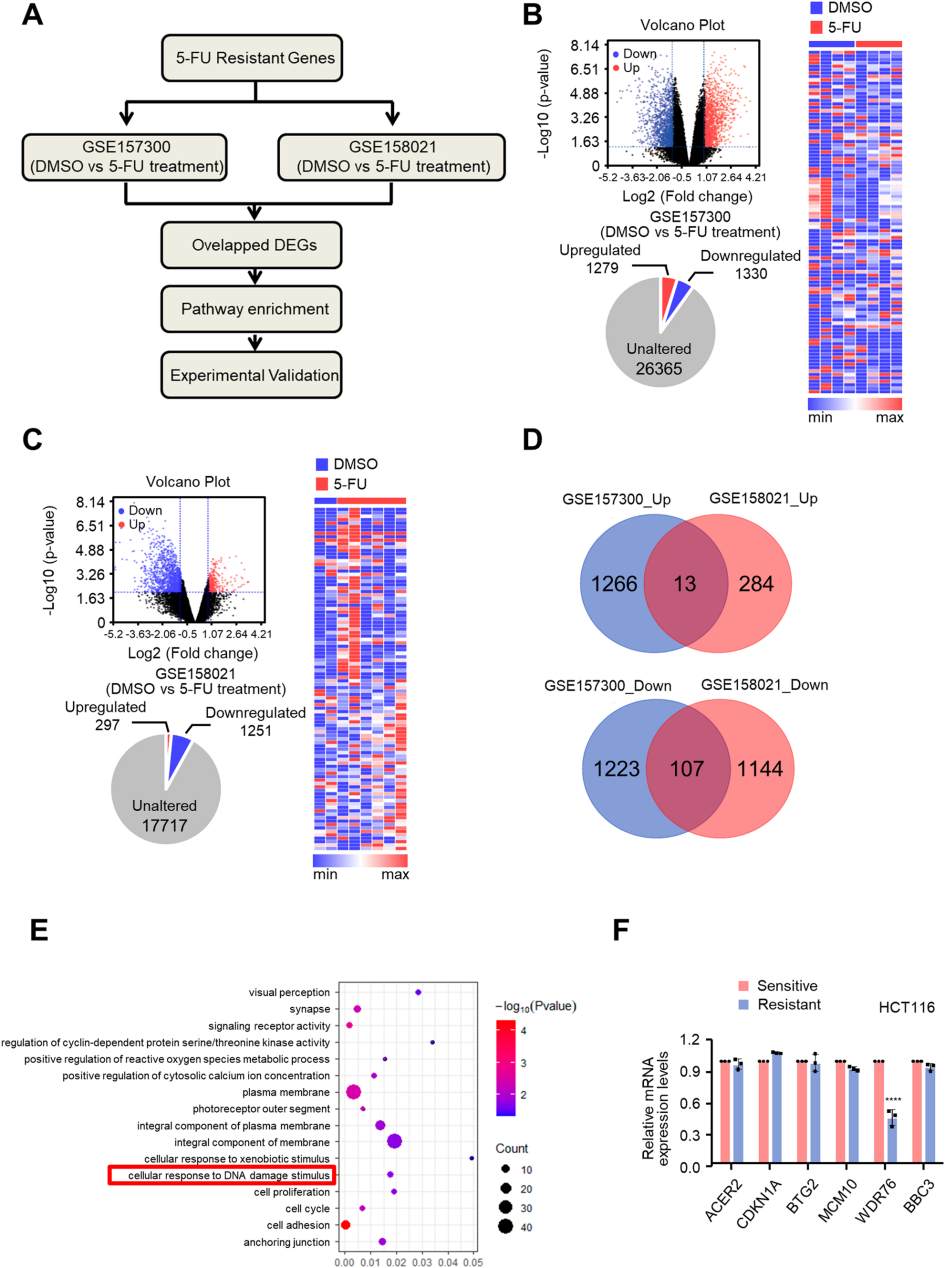
WDR76 is screened and verified in 5-FU resistant colon cancer. **A** Screening flowchart. **B**, **C** A volcano plot illustrating DEGs between DMSO and 5-FU treated HCT116 cells in GSE157300 or GSE158021. Values are presented as the log10 of tag counts. Pie chart revealed 1279 genes were upregulated and 1330 genes downregulated in GSE157300 and 297 genes were upregulated and 1251 genes downregulated in GSE158021. The hierarchical clustering of the RNA-seq analysis results shows all genes that were significantly differently expressed. **D** Venn analysis of the DEGs. The middle part represented the intersection of the results. **E** GO analysis based on the overlapped 120 DEGs. **F** Expressions of ACER2, CDKN1A, BTG2, MCM10, WDR76, BBC3 in HCT116-S and HCT116-R cell lines were performed by quantitative real-time PCR. All values displayed are mean ± SD and have been duplicated 3 times with similar results. All the experiments are repeated at least three times; ****P < 0.0001

To screen out the regulatory factors related to 5-FU resistance in colon cancer, the 5-FU related data from the GEO database were analyzed according to the flow chart in Fig. 1A. In GSE157300 dataset, 1279 upregulated and 1330 downregulated genes compared with 5-FU treated HCT116 cells were selected in the parent cells (Fig. 1B), and 297 upregulated and 1251 downregulated genes from GSE158021 dataset (Fig. 1C). After overlapping the above two analysis results, 13 upregulated and 107 downregulated genes finally obtained (Fig. 1D), and employed for the enrichment analysis, which finally identified 16 significant pathways (Fig. 1E). In addition, 6 genes were surprisingly found enriched in the pathway related to cellular response to DNA damage stimulus, that were, cyclin dependent kinase inhibitor 1A (CDKN1A), alkaline ceramidase 2 (ACER2), BTG anti-proliferation factor 2 (BTG2), minichromosomal maintenance 10 replication initiation factor (MCM10), WD repeat domain 76 (WDR76) and BCL2 binding component 3 (BBC3). Subsequently, the mRNA level of the above 6 genes in HCT116 sensitive (HCT116-S) and resistant (HCT116-R) cells was identified, with the significantly decreased expression of WDR76 in HCT116-R cells compared to HCT116-S cells, without significant differences among the other 5 genes in these two cells (Fig. 1F). Therefore, it was speculated that WDR76 may be related to the resistance of colon cancer to 5-FU.

### WDR76 regulates the sensitivity of resistant colon cancer cells to 5-FU

**Fig. 2:**
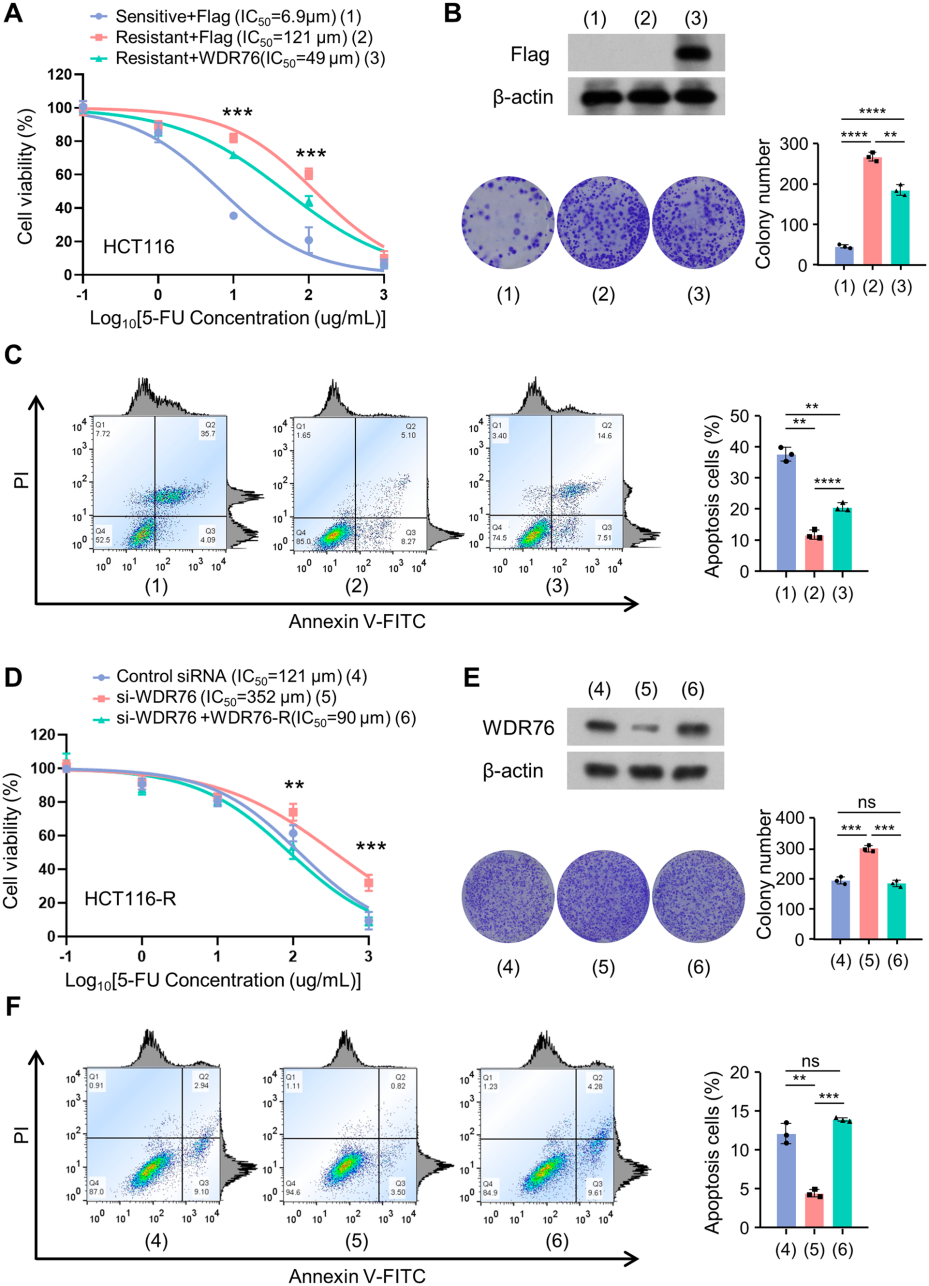
WDR76 regulates the sensitivity of resistant colon cancer cells to 5-FU. **A** Cell viability assays of sensitive HCT116 cells to 5-FU (HCT116-S) and resistant HCT116 cells to 5-FU (HCT116-R) transfected with empty vector or Flag-WDR76 and treated with 5-FU at gradient concentrations. **B**, **C** Colony formation and cell apoptosis of cells transfected as (**A**) and treated with 20 μm 5-FU. (D) Cell viability assays of HCT116-R transfected with control siRNA or si-WDR76 or si-WDR76 plus WDR76 re-expression (WDR76-R) and treated with 5-FU at gradient concentrations. **E**, **F** Colony formation and cell apoptosis of cells transfected as (**D**) and treated with 20 μm 5-FU. Illustrative images show colonies in plates. Histograms show colony number. Cell apoptosis were evaluated by flow cytometry and the apoptotic cell percentage was statistically analyzed. All values displayed are mean ± SD and have been duplicated 3 times with similar results. All the experiments are repeated at least three times; **P < 0.01, ***P < 0.001, ****P < 0.0001

By measuring the survival of colon cancer cell exposed to 5-FU, the IC_50_ (half maximal inhibitory concentration) of HCT116-S cells was 6.9 μm, which was significantly lower compared to HCT116-R cells of 121 μm. The overexpressed WDR76 in HCT116-R cells reduced the IC_50_ to 49 μm (Fig. 2A). Exposed to 5-FU, colony formation and apoptosis assays demonstrated the higher viability of HCT116-R cells compared to HCT116-S cells, while the WDR76 overexpression reduced the colony numbers and increased the apoptosis rate in HCT116-R cells (Fig. 2B, C). The same results were also found in COLO320-S and COLO320-R cells (Fig. S1A-C).

Next, the WDR76 siRNA was transfected into HCT116-R cells, and the IC_50_ was observed increased from 121 to 352 μm, which was reversed by the expression of WDR76, as of 90 μm. WDR76 knockdown increased the viability of HCT116-R cells. Meanwhile, the colony formation and apoptosis assays also demonstrated the higher viability of cells with WDR76 knockdown, which was reversed by the expression of WDR76 (Fig. 2D–F). These outcomes indicated that WDR76 could regulate the sensitivity of resistant colon cancer cells to 5-FU.

### WDR76 upregulates the sensitivity of resistant colon cancer cells to 5-FU by promoting the degradation of HRAS

**Fig. 3:**
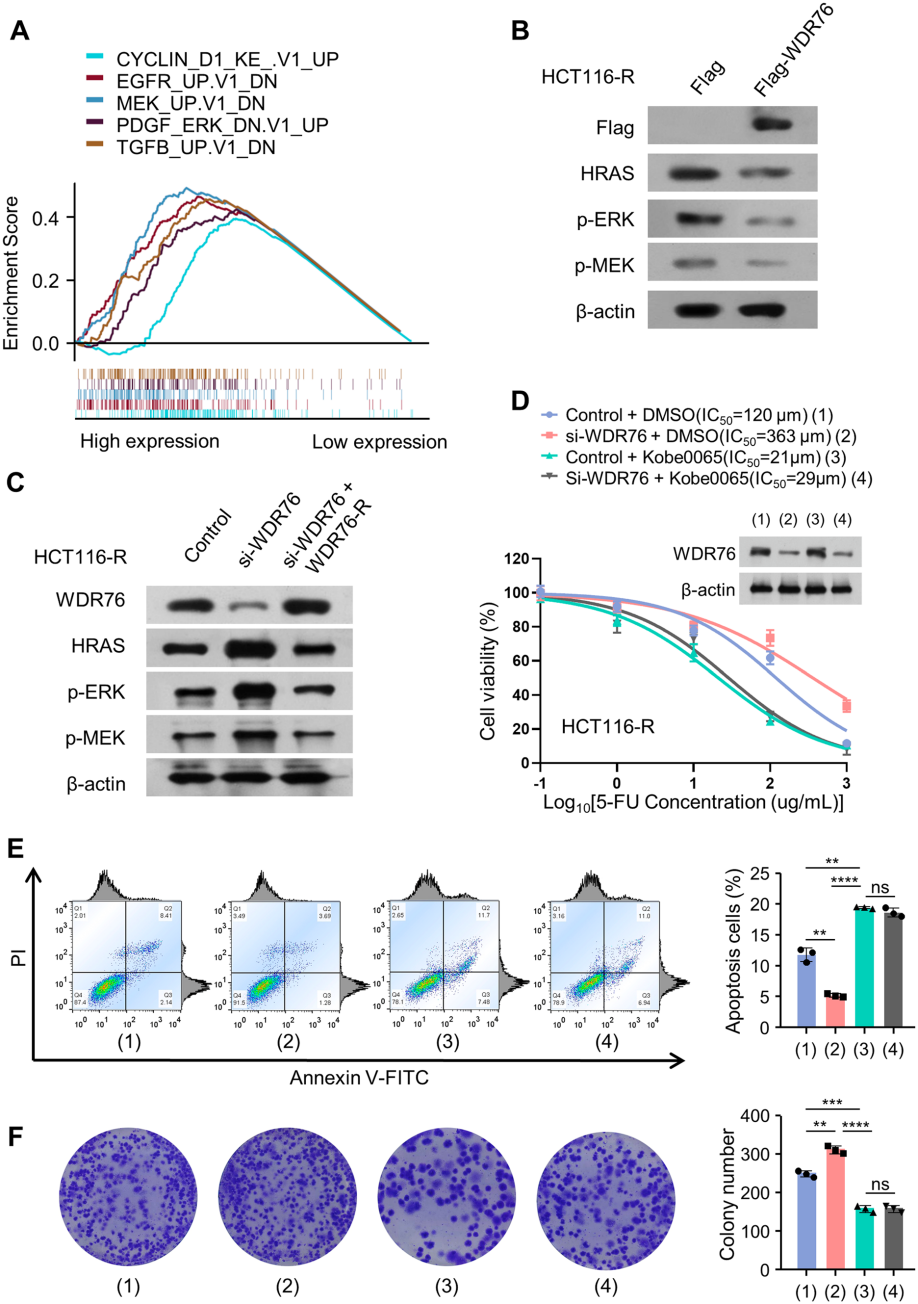
WDR76 upregulates the sensitivity of resistant colon cancer cells to 5-FU by promoting the degradation of HRAS. **A** GSEA analysis was used to analyze the pathways correlated with WDR76. **B**, **C** Westernblot was applied to analyze the expressions of HRAS related proteins in resistant HCT116 cells to 5-FU (HCT116-R) transfected into Flag or Flag-WDR76 and control or si-WDR76 or si-WDR76 plus WDR76 re-expression (WDR76-R). **D** Cell viability assays of HCT116-R cells transfected with control or si-WDR76 and treated with DMSO or Kobe0065. **E**, **F** Colony formation and cell apoptosis of cells transfected as (**D**) and treated with 20 μm 5-FU. Illustrative images show colonies in plates. Histograms show colony number. Cell apoptosis were evaluated by flow cytometry and the apoptotic cell percentage was statistically analyzed. All values displayed are mean ± SD and have been duplicated 3 times with similar results. All the experiments are repeated at least three times; **P < 0.01, ***P < 0.001, ****P < 0.0001

To further explore the mechanism of WDR76 to reverse the sensitivity of colon cancer resistant to 5-FU, the correlation between WDR76 and pathways in TCGA-COAD dataset was analyzed using Gene Set Enrichment Analysis (GSEA). It was surprising to demonstrate the correlation of the higher expressed WDR76 with 5 pathways, including CYCLIN_D1_KE_.V1_UP, EGFR_UP.V1_DN, MEK_UP.V1_DN, PDGF_ERK_DN.V1_UP and TGFB_UP.V1_DN. Importantly, these are upstream or downstream pathways related to HRAS (Fig. 3A). Furthermore, the WDR76 was overexpressed on HRAS, p-ERK and p-MEK in HCT116-R cells, which downregulated the protein levels of HRAS, p-ERK and p-MEK (Fig. 3B), and reversed by WDR76 knockdown, while was reversed again by reexpression of WDR76 (Fig. 3C). Kobe0065 as an inhibitor of the interaction between HRAS and cRaf could effectively inhibit the function of HRAS, which was revealed to abrogate the alterations of IC50 and cell viability due to the WDR76 knockdown in HCT116-R cells (Fig. 3D), as consistently demonstrated by cell apoptosis and clone formation (Fig. 3E, F). The above results were also verified in COLO320-R cells (Fig. S2A–D). To sum up, WDR76 could affects the resistance of 5-FU by manipulating HRAS in colon cancer.

### WDR76 knockdown decreases 5-FU sensitivity of colon cancer cells through HRAS in vivo

**Fig. 4:**
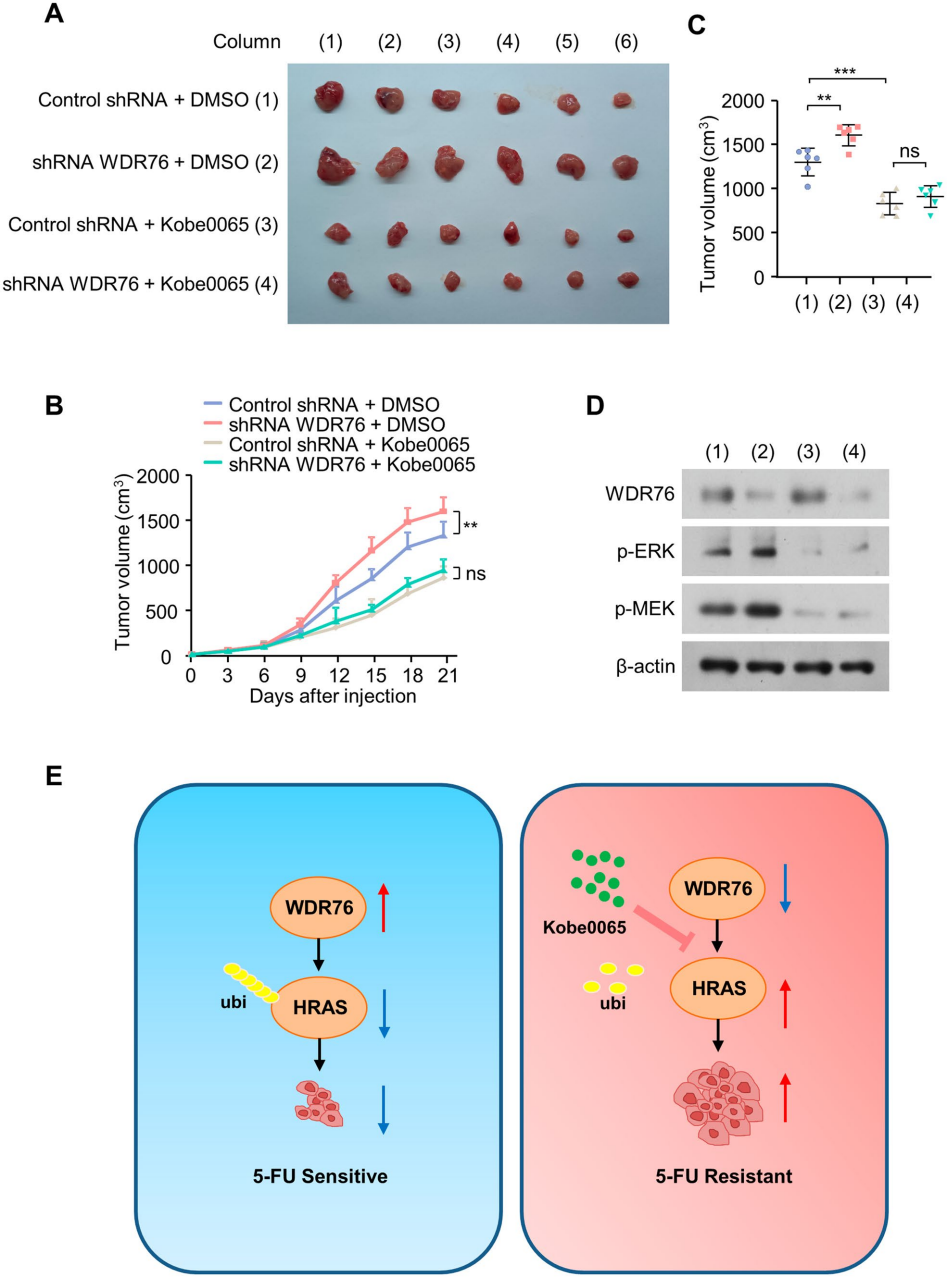
WDR76 knockdown decreases 5-FU sensitivity of colon cancer cells through HRAS in vivo. **A**–**C** Resistant HCT116 cells to 5-FU stably expressing control shRNA or WDR76 shRNA were subcutaneously injected into the left flanks of 6-week female nude mice. Each group was injected with 5-FU (10 mg/kg) or normal saline once a week after 7 days. All mice were treated with 5-FU every 3 days after injected by cancer cells for 7 days. The volume of the tumors was measured at the interval of three days. **D** Westernblot of the representative tumor tissues to detect the expression of HRAS related proteins. **E** Schematic of the regulatory network in this study

HCT116-R cells harbored by WDR76 shRNA or control shRNA were injected subcutaneously into the left flanks of 6-week female nude mice. In 7 days, normal saline or Kobe0065 was injected once a week. All mice were treated with 5-FU every 3 days after injected by cancer cells for 7 days. The results revealed the significantly stronger tumorigenicity of WDR76 stable knockdown cells compared to the control shRNA, which was abolished by using the HRAS specific inhibitor Kobe0065 (Fig. 4A–C). Higher p-ERK and p-MEK were expressed in tumors with WDR76 shRNA, which were weakened by Kobe0065 (Fig. 4D). These results demonstrated the reduced sensitivity of colon cancer cells to 5-FU by WDR76 knockdown through HRAS in vivo.

## Discussion

Chemotherapy serves as the first choice for medical treatment of tumors in most cases [20–22], where 5-FU is an important drug for many chemotherapy regimens, including DCF, FOLFIRI, ECF, etc. FOLFOX is employed as a first-line chemotherapy regimen for colon cancer [23]. However, despite the wide clinical application, the resistance of 5-FU remains a major challenge in the treatment, which will influence the life time and the quality of life [24, 25]. Gao et al. demonstrated the inhibition of EGFR could effectively enhance the sensitivity of colon cancer cells to 5-FU [26]. MicroRNA-122 could re-sensitize the 5-FU-resistant colon cancer cells to 5-FU by inhibiting PKM2 [27]. Nevertheless, the mechanism of this resistance in the treatment of colon cancer still requires to be explored. In the present study, the differentially expressed genes in 5-FU resistant and sensitive cells analyzed. Finally, 6 genes that may involve in the biological process of 5-FU resistance were obtained using the pathway enrichment. The significant overexpressed WDR76 in resistant cells was identified by qRT-PCR. Further experiments of cell apoptosis and clone formation revealed the increased sensitivity of resistant cells to 5-FU by WDR76. Briefly, it was the first research to clarify the molecular mechanism of WDR76 reversing 5-FU resistance in colon cancer.

Up to now, the research of WDR76 on cancer regulation is mainly focused on the occurrence and development of liver cancer and colorectal carcinoma. For example, WDR76 has been demonstrated to play a role as an E3 linker protein to mediate the polyubiquitination-dependent degradation of RAS, leading to the inhibition of proliferation, transformation, and invasion of liver cancer cells. In colorectal carcinoma, WDR76 could degrade pan-RAS and inhibit cancer stem cell activation, as well as the progression of colorectal carcinoma cell cycle by degrading KRAS, thereby damaging the tumor development. By bioinformatics research, WDR76 also participates in the manipulation of immune invasion of lung adenocarcinoma, exerting a predictive value for the prognosis of lung adenocarcinoma [28]. However, there report few studies of WDR76 in drug resistance to chemotherapy.

Based on the issue of resistance to 5-FU in the treatment of colon cancer, it is urgently required to develop the effective strategies. HRAS, a member of RAS protein family [29–31], has not been reported in 5-FU resistant colon cancer. Our study indicated that WDR76/HRAS axis could reversely manipulate the sensitive of resistant colon cancer cells to 5-FU. This work enriched the molecular regulatory network of colon cancer cells resistant to 5-FU, providing a new strategy for further research on drug resistance.

## Conclusions

In brief, this work revealed the effect of WDR76 on 5-FU resistance of colon cancer cells in vivo and in vitro. WDR76 partly restores 5-FU resistance by downregulating the level of HRAS. The WDR76/HRAS axis might serve as an effective target of 5-FU resistance in colon cancer. Whether WDR76 could promote the sensitive of colon cancer to other chemotherapy drugs remains to be further verified. This study provides a new target and direction for studying 5-FU resistance. However, the function of WDR76 in human tumor tissue still needs further research.

## Supplementary Information

Supplementary Figure 1. (A) Cell viability assays of sensitive COLO320 cells to 5-FU (COLO320-S) and resistant COLO320 cells to 5-FU (COLO320-R) transfected with empty vector or Flag-WDR76 and treated with 5-FU at gradient concentrations. (B, C) Colony formation and cell apoptosis of cells transfected as (A) and treated with 20μm 5-FU. Illustrative images show colonies in plates. Histograms show colony number. Cell apoptosis were evaluated by flow cytometry and the apoptotic cell percentage was statistically analyzed. All values displayed are mean ± SD and have been duplicated 3 times with similar results. All the experiments are repeated at least three times; **P < 0.01, ***P < 0.001, ****P < 0.0001. Supplementary file1 (TIF 1292 KB).

Supplementary Figure 2. (A, B) Westernblot was applied to analyze the expressions of HRAS related proteins in resistant COLO320 cells to 5-FU (COLO320-R) transfected into Flag or Flag-WDR76 and control or si-WDR76 or si-WDR76 plus WDR76 re-expression (WDR76-R). (C) Cell viability assays of COLO320-R cells transfected with control or si-WDR76 and treated with DMSO or Kobe0065. (D, E) Colony formation and cell apoptosis of cells transfected as (C) and treated with 20μm 5-FU. Illustrative images show colonies in plates. Histograms show colony number. Cell apoptosis were evaluated by flow cytometry and the apoptotic cell percentage was statistically analyzed. All values displayed are mean ± SD and have been duplicated 3 times with similar results. All the experiments are repeated at least three times; **P < 0.01, ***P < 0.001, ****P < 0.0001. Supplementary file2 (TIF 2008 KB).

## Data Availability

All data generated or analysed during this study are included in this article and its additional information files.

## References

[1] Fleming CA, O'Connell EP, Kavanagh RG (2021). Body composition, inflammation, and 5-year outcomes in colon cancer. JAMA Netw Open.

[2] Teufel A, Gerken M, Fürst A (2020). Benefit of adjuvant chemotherapy in high-risk colon cancer: a 17-year population-based analysis of 6131 patients with Union for International Cancer Control stage II T4N0M0 colon cancer. Eur J Cancer.

[3] Angell HK, Bruni D, Barrett JC (2020). The immunoscore: colon cancer and beyond. Clin Cancer Res.

[4] Schilsky RL (2018). A new IDEA in adjuvant chemotherapy for colon cancer. N Engl J Med.

[5] Gunjur A (2018). Short vs long course adjuvant chemotherapy for colon cancer. Lancet Oncol.

[6] Manjelievskaia J, Brown D, McGlynn KA (2017). Chemotherapy use and survival among young and middle-aged patients with colon cancer. JAMA Surg.

[7] Zaborowski AM, Abdile A (2021). Characteristics of early-onset vs late-onset colorectal cancer: a review. JAMA Surg.

[8] Sica GS, Vinci D, Siragusa L (2023). Definition and reporting of lymphadenectomy and complete mesocolic excision for radical right colectomy: a systematic review. Surg Endosc.

[9] Moehler M, Maderer A, Thuss-Patience PC (2020). Cisplatin and 5-fluorouracil with or without epidermal growth factor receptor inhibition panitumumab for patients with non-resectable, advanced or metastatic oesophageal squamous cell cancer: a prospective, open-label, randomised phase III AIO/EORTC trial (POWER). Ann Oncol.

[10] Lamarca A, Palmer DH, Wasan HS (2021). Second-line FOLFOX chemotherapy versus active symptom control for advanced biliary tract cancer (ABC-06): a phase 3, open-label, randomised, controlled trial. Lancet Oncol.

[11] Sauraj, Kumar SU, Gopinath P (2017). Synthesis and bio-evaluation of xylan-5-fluorouracil-1-acetic acid conjugates as prodrugs for colon cancer treatment. Carbohydr Polym.

[12] Hong YS, Nam BH, Kim KP (2014). Oxaliplatin, fluorouracil, and leucovorin versus fluorouracil and leucovorin as adjuvant chemotherapy for locally advanced rectal cancer after preoperative chemoradiotherapy (ADORE): an open-label, multicentre, phase 2, randomised controlled trial. Lancet Oncol.

[13] Stein A, Hiemer S, Schmoll HJ (2011). Adjuvant therapy for early colon cancer: current status. Drugs.

[14] Yang J, Wang F, Chen B (2021). The role of WDR76 protein in human diseases. Bosn J Basic Med Sci.

[15] Gilmore JM, Sardiu ME, Groppe BD (2016). WDR76 Co-localizes with heterochromatin related proteins and rapidly responds to DNA damage. PLoS ONE.

[16] Park JC, Jeong WJ, Seo SH (2019). WDR76 mediates obesity and hepatic steatosis via HRas destabilization. Sci Rep.

[17] Jeong WJ, Park JC, Kim WS (2019). WDR76 is a RAS binding protein that functions as a tumor suppressor via RAS degradation. Nat Commun.

[18] Ro EJ, Cho YH, Jeong WJ (2019). WDR76 degrades RAS and suppresses cancer stem cell activation in colorectal cancer. Cell Commun Signal.

[19] Park JY (2019). New concept of hepatocellular carcinoma treatment with the tumor suppressor ‘WDR76’ through ‘RAS’ degradation. Korean J Gastroenterol.

[20] Plava J, Cihova M, Burikova M (2019). Recent advances in understanding tumor stroma-mediated chemoresistance in breast cancer. Mol Cancer.

[21] Rodriguez-Brenes IA, Kurtova AV, Lin C (2017). Cellular hierarchy as a determinant of tumor sensitivity to chemotherapy. Cancer Res.

[22] D'Alterio C, Scala S, Sozzi G (2020). Paradoxical effects of chemotherapy on tumor relapse and metastasis promotion. Semin Cancer Biol.

[23] Tsai YJ, Lin JK, Chen WS (2016). Adjuvant FOLFOX treatment for stage III colon cancer: how many cycles are enough?. Springerplus.

[24] Qin Y, Ma X, Guo C (2022). MeCP2 confers 5-fluorouracil resistance in gastric cancer via upregulating the NOX4/PKM2 pathway. Cancer Cell Int.

[25] Pranzini E, Pardella E, Muccillo L (2022). SHMT2-mediated mitochondrial serine metabolism drives 5-FU resistance by fueling nucleotide biosynthesis. Cell Rep.

[26] Gao SJ, Ren SN, Liu YT (2021). Targeting EGFR sensitizes 5-Fu-resistant colon cancer cells through modification of the lncRNA-FGD5-AS1-miR-330-3p-hexokinase 2 axis. Mol Ther Oncolytics.

[27] He J, Xie G, Tong J (2014). Overexpression of microRNA-122 re-sensitizes 5-FU-resistant colon cancer cells to 5-FU through the inhibition of PKM2 in vitro and in vivo. Cell Biochem Biophys.

[28] Fang L, Yu G, Yu W (2021). The correlation of WDR76 expression with survival outcomes and immune infiltrates in lung adenocarcinoma. PeerJ.

[29] Bièche I, Coussy F, El-Botty R (2021). HRAS is a therapeutic target in malignant chemo-resistant adenomyoepithelioma of the breast. J Hematol Oncol.

[30] Topf MC, Wang ZX, Tuluc M (2018). TERT, HRAS, and EIF1AX mutations in a patient with follicular adenoma. Thyroid.

[31] Chen YT, Huang ZY, Tang HH (2020). Pterostilbene sensitizes cisplatin-resistant human bladder cancer cells with oncogenic HRAS. Cancers.

