# Supplementary figures and images for: WDR76 regulates 5-fluorouracil sensitivity in colon cancer via HRAS
Source: Discov Oncol. 2023 Apr 20;14:45. doi: 10.1007/s12672-023-00656-9 (PMC10119360; doi:10.1007/s12672-023-00656-9)

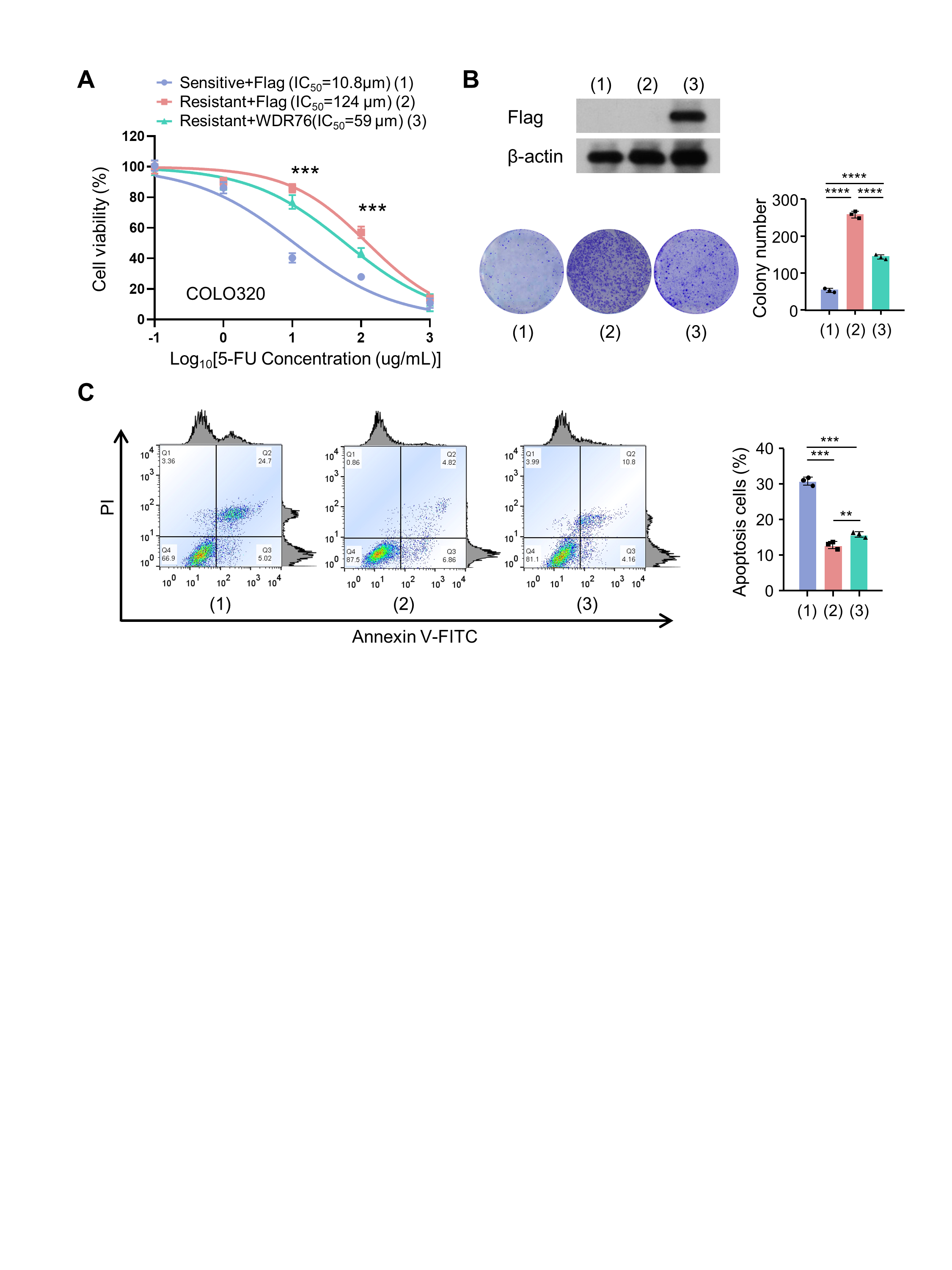

Supplement: Supplementary file 1 — Supplementary Figure 1. (A) Cell viability assays of sensitive COLO320 cells to 5-FU (COLO320-S) and resistant COLO320 cells to 5-FU (COLO320-R) transfected with empty vector or Flag-WDR76 and treated with 5-FU at gradient concentrations. (B, C) Colony formation and cell apoptosis of cells transfected as (A) and treated with 20μm 5-FU. Illustrative images show colonies in plates. Histograms show colony number. Cell apoptosis were evaluated by flow cytometry and the apoptotic cell percentage was statistically analyzed. All values displayed are mean ± SD and have been duplicated 3 times with similar results. All the experiments are repeated at least three times; **P < 0.01, ***P < 0.001, ****P < 0.0001. Supplementary file1 (TIF 1292 KB). [file 12672_2023_656_MOESM1_ESM.tif]

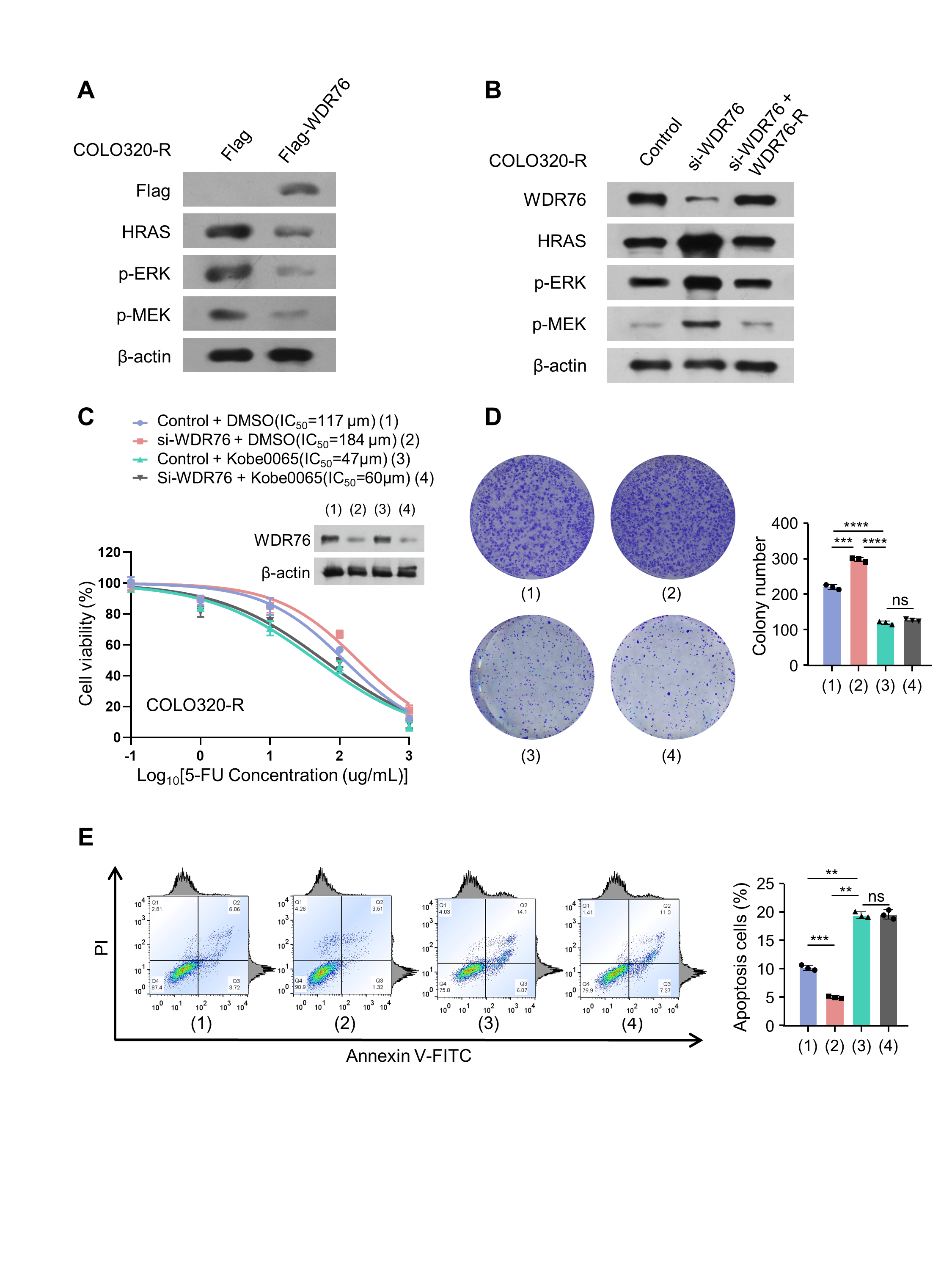

Supplement: Supplementary file 2 — Supplementary Figure 2. (A, B) Westernblot was applied to analyze the expressions of HRAS related proteins in resistant COLO320 cells to 5-FU (COLO320-R) transfected into Flag or Flag-WDR76 and control or si-WDR76 or si-WDR76 plus WDR76 re-expression (WDR76-R). (C) Cell viability assays of COLO320-R cells transfected with control or si-WDR76 and treated with DMSO or Kobe0065. (D, E) Colony formation and cell apoptosis of cells transfected as (C) and treated with 20μm 5-FU. Illustrative images show colonies in plates. Histograms show colony number. Cell apoptosis were evaluated by flow cytometry and the apoptotic cell percentage was statistically analyzed. All values displayed are mean ± SD and have been duplicated 3 times with similar results. All the experiments are repeated at least three times; **P < 0.01, ***P < 0.001, ****P < 0.0001. Supplementary file2 (TIF 2008 KB). [file 12672_2023_656_MOESM2_ESM.tif]
